# A quality-comprehensive-evaluation-index-based model for evaluating traditional Chinese medicine quality

**DOI:** 10.1186/s13020-023-00782-0

**Published:** 2023-07-28

**Authors:** Jia Chen, Lin-Fu Li, Zhao-Zhou Lin, Xian-Long Cheng, Feng Wei, Shuang-Cheng Ma

**Affiliations:** 1 Institute for Control of Chinese Traditional Medicine and Ethnic Medicine (ICCTMEM), National Institutes for Food and Drug Control (NIFDC), No. 31, Huatuo Road, Daxing District, Beijing, 102629 China; 2 College of Pharmacy, Gannan Medical University, No. 1, Yixueyuan Road, Zhanggong District, Ganzhou, 341000 China; 3 Fengtai District, Beijing Tongrentang Technology Development Co., Ltd., No. 20, Nansanhuan Zhonglu Road, Beijing, 100075 China

**Keywords:** Licorice, Quality grading, Multiweighted quality attribute index, Chemometrics, Traditional Chinese medicine, Quality comprehensive evaluation index, Quality control, Pharmacological activity-based quality index

## Abstract

**Background:**

Evaluating traditional Chinese medicine (TCM) quality is a powerful method to ensure TCM safety. TCM quality evaluation methods primarily include characterization evaluations and separate physical, chemical, and biological evaluations; however, these approaches have limitations. Nevertheless, researchers have recently integrated evaluation methods, advancing the emergence of frontier research tools, such as TCM quality markers (Q-markers). These studies are largely based on biological activity, with weak correlations between the quality indices and quality. However, these TCM quality indices focus on the individual efficacies of single bioactive components and, therefore, do not accurately represent the TCM quality. Conventionally, provenance, place of origin, preparation, and processing are the key attributes influencing TCM quality. In this study, we identified TCM-attribute-based quality indices and developed a comprehensive multiweighted multi-index-based TCM quality composite evaluation index (QCEI) for grading TCM quality.

**Methods:**

The area of origin, number of growth years, and harvest season are considered key TCM quality attributes. In this study, licorice was the model TCM to investigate the quality indicators associated with key factors that are considered to influence TCM quality using multivariate statistical analysis, identify biological-evaluation-based pharmacological activity indicators by network pharmacology, establish real quality indicators, and develop a QCEI-based model for grading TCM quality using a machine learning model. Finally, to determine whether different licorice quality grades differently reduced the inflammatory response, TNF-α and IL-1β levels were measured in RAW 264.7 cells using ELISA analysis.

**Results:**

The 21 quality indices are suitable candidates for establishing a method for grading licorice quality. A computer model was established using SVM analysis to predict the TCM quality composite evaluation index (TCM QCEI). The tenfold cross validation accuracy was 90.26%. Licorice diameter; total flavonoid content; similarities of HPLC chromatogram fingerprints recorded at 250 and 330 nm; contents of liquiritin apioside, liquiritin, glycyrrhizic acid, and liquiritigenin; and pharmacological activity quality index were identified as the key indices for constructing the model for evaluating licorice quality and determining which model contribution rates were proportionally weighted in the model. The ELISA analysis results preliminarily suggest that the inflammatory responses were likely better reduced by premium-grade than by first-class licorice.

**Conclusions:**

In the present study, traditional sensory characterization and modern standardized processes based on production process and pharmacological efficacy evaluation were integrated for use in the assessment of TCM quality. Multidimensional quality evaluation indices were integrated with a machine learning model to identify key quality indices and their corresponding weight coefficients, to establish a multiweighted multi-index and comprehensive quality index, and to construct a QCEI-based model for grading TCM quality. Our results could facilitate and guide the development of TCM quality control research.

**Supplementary Information:**

The online version contains supplementary material available at 10.1186/s13020-023-00782-0.

## Background

Quality control (QC) is a powerful method for ensuring the quality and safety of traditional Chinese medicine (TCM), and strengthening TCMQC is crucial to maximize the quality, safety, and effectiveness of TCM and promote the development of high-quality industrial TCM applications. As the main TCMQC research focus, evaluating TCM quality is a powerful method for determining TCM quality and safety. In China, because TCM quality has been conventionally evaluated using Chinese-Pharmacopoeia-based national drug standards, most TCM quality can only meet the minimum standards; specifically, although TCM can be authenticated, TCM advantages and disadvantages could not be distinguished. Consequently, TCM quality is inconsistent, high quality and affordable prices are both difficult to achieve, and market fairness is severely hindered, which greatly limits the development of high-quality industrial TCM applications. TCM grading is the main method for determining TCM quality and is based on TCM commodity specifications graded according to previous dynastic practical experience. For thousands of years, TCM materials have originated from wild sources. However, with the depletion of wild resources in recent years, artificially planted TCMs have become widespread in the market. Consequently, the original classification methods are not practical, with poor correlation between traditional TCM classification and current TCM quality. Similarly, appropriate evaluation indices that reliably reflect the internal quality of TCM currently available in the market, as well as the contributions of different quality indices, have yet to be formulated.

Recently, many research teams have comprehensively evaluated TCM quality. Some teams have combined multiple chemical component indices and multivariate statistical analysis to biologically evaluate TCM quality [1, 2]. This approach solves the key problem wherein evaluating TCM quality cannot accurately reflect TCM clinical efficacy or safety.

Furthermore, this approach addresses the shortcomings of chemical and characterization evaluation approaches. However, this method does not consider the influence of area or origin and standardization on the quality. Some teams have proposed a Chinese medicine quality marker (Q-marker)-based model for evaluating TCM quality. Establishing Q-markers integrates multiple disciplines, such as natural product and analytical chemistries, bionics, chemometrics, pharmacology, systems biology, and pharmacodynamics. Q-marker-based fingerprinting and multicomponent determination have led to the development of more scientific TCMQC systems [3–5]. However, no effective programs or methods have yet been developed for identifying Q-markers.

Conventionally, provenance, place of origin, machining, and processing are the key attributes determining the TCM quality. Because TCM quality originates from production, the genuine production area and standardization (which involve traditional excavation and growth for a sufficient number of years) are important attributes ensuring and stabilizing the TCM quality.

In this study, authentic licorice (*Glycyrrhiza uralensis* Fisch.) was grown using standardized production methods to ensure a high-quality TCM product [6]. Key quality attributes of licorice, including the genuine production area, growth years, and harvest season, were used to identify internal quality indicators highly related to quality characteristics and to biologically evaluate pharmacological activity indicators. The key quality attributes and their corresponding weighted contributions were established through multidimensional research. Finally, a method for grading TCM quality was established based on the proposed quality composite evaluation index (QCEI). To achieve this objective, we collected 282 batches of licorice samples that differed in the production area, growth years, and harvest season.

The proposed method of grading TCM quality is important for developing genuine bases for growing TCM plants, standardizing TCM planting conditions, strengthening QC for the entire TCM production process, and ensuring a standardized and orderly TCM market.

## Methods

### Reagents and chemicals

Acetonitrile and phosphoric acid of high-performance liquid chromatography (HPLC) grade were supplied by Fisher Scientific (Fairlawn, NJ, USA). Ultrapure water (18.2 MΩ) was prepared using a Milli-Q™ water-purification system (Millipore, Milford, MA, USA). All the other reagents were analytical grade and were purchased from Sinopharm Chemical Reagents (Shanghai, China).

The reference compounds liquiritin (number 1; lot number111610-201908; 95.00% pure), formononetin (2; 111703–201504; 95.00%), and glycyrrhizic acid monoammonium salt (3; 110731–202021; 96.20%) were supplied by the National Institutes for Food and Drug Control (NIFDC; Beijing, China).

Reference compounds liquiritigenin (4; PRF20042742; 99.50%), isoliquiritigenin (5; PRF20060943; 99.87%), isoliquiritin (6; PRF20040923; 98.23%), neoisoliquiritin (7; PRF20060942; 99.25%), glycycoumarin (8; PRF20060921; 99.77%), isoliquiritin apioside (9; PRF9101021; 97.04%), liquiritin apioside (10; PRF9050224; 99.95%), and violanthin (11; PRF9110601; 95.77%) were purchased from Chengdu Biopurify Phytochemicals, Ltd. (Chengdu, China).

Reference compounds licoisoflavone B (12; PS20110202; 98.02%), glycyrol (13; PS010089; 98.71%), and licoisoflavone A (14; PS010124; 98.46%) were obtained from Chengdu Push Biotech (Chengdu, China).

Reference compounds licoflavonol (15; MUST-20041311; 98.86%) and semilicoisoflavone B (16; MUST20072104; 99.91%) were purchased from Chengdu Must Biotechnology (Chengdu, China).

Licoricesaponin G2 (17; PCS200904; 98.59%) was purchased from Chengdu PureChem-Standard Biotech Co., Ltd. (Chengdu, China).

Licorice samples (282 batches) were collected from Gansu (S1–134), Inner Mongolia (S135–255), and Xinjiang (S256–282) in China. Representative licorice samples were collected from traditional licorice-producing areas: Yuzhong in Gansu Province (S51–90), Bayannao’er in Inner Mongolia (S145–184) and Hangjin Banner in Inner Mongolia (S185–225). These samples had continuously grown for 2–5 years in the same planting area and were harvested in the same season to investigate how the number of growth years affected the licorice quality. To investigate how the traditional genuine and nongenuine growing areas affected licorice quality, licorice samples were collected from traditional genuine growing areas in Inner Mongolia and Gansu and nongenuine growing areas in the main Xinjiang production area. The traditional genuine growing area samples were identified as S41–50, S71–90, S135–144, S175–184, and S214–225, while the nongenuine growing area samples were identified as S256–282. All samples were used to investigate how the production area affected the quality of licorice grown for the same number of years and harvested in the same season. To investigate how the harvest season affected the licorice quality, samples of licorice planted in the same location and grown for the same number of years were collected in spring and autumn. Autumn samples, identified as S41–50, S155–164, and S214–225, were harvested in November 2018 and 2019; spring samples, identified as S31–40, S236–245, and S246–255, were harvested in March 2018 and 2020. The sample origins, number of growth years, and harvest times are summarized in Additional file 1: Table S1. The licorice samples were authenticated as dried *Glycyrrhiza uralensis* Fisch. ex DC. (Chinese licorice) roots by Professor Nanping Zhang (NIFDC, Beijing, China). Voucher specimens were deposited in the Museum of Chinese Traditional Drugs at NIFDC [7].

### Preparation of the standard reference and sample solutions

#### Preparation of standard reference solutions for HPLC–photodiode-array (PDA) detection

Standard reference solutions were prepared by completely dissolving compounds (1)–(17) in 70% aqueous methanol to obtain the following concentrations (µg/mL): (1) 90.44, (2) 4.16, (3) 182.20, (4) 15.70, (5) 4.62, (6) 19.96, (7) 11.36, (8) 8.06, (9) 36.54, (10) 116.05, (11) 5.47, (12) 9.17, (13) 14.00, (14) 3.54, (15) 4.40, (16) 11.63, and (17) 46.26. All solutions were stored at 4 °C prior to the analyses.

#### Preparation of the standard reference solution for ultraviolet–visible (UV–vis) spectroscopy

Liquiritin was completely dissolved in 70% aqueous methanol to prepare a 22.53 µg/mL standard reference solution; this solution was stored at 4 °C prior to the analyses.

#### Solution preparation for standardizing (UV–vis) absorption spectra

Different reference stock solution volumes (0.2, 0.5, 1.0, 2.0, 3.0, 4.0, or 5.0 mL) were accurately pipetted and added to 10 mL volumetric flasks fitted with rubber stoppers. Then, 0.5 mL of 10% KOH solution was pipetted, added to a corresponding volumetric flask, left at 25 °C for 5 min, diluted with 70% ethanol, and shaken well.

#### Preparation of the sample solutions for HPLC–PDA detection

All the samples were pulverized and screened through a 50-mesh sieve. Dried powder samples (1.0 g) were accurately weighed using an XSE 205DU electronic balance (Mettler Toledo, Zurich, Switzerland) and placed in 100 mL conical flasks fitted with rubber stoppers. The compounds were extracted via ultrasonication in 50 mL of 70% aqueous methanol for 0.5 h. The mixtures were centrifuged at 12,000 rpm for 10 min at room temperature. Finally, the supernatants were filtered through a 0.22-μm membrane prior to injection into an HPLC–PDA detector.

#### Preparation of the sample solutions for UV–vis spectroscopy

All the samples were pulverized and screened through a 50-mesh sieve. Dried powder samples (0.2 g) were accurately weighed and placed in 50 mL conical flasks fitted with rubber stoppers. The compounds were extracted via ultrasonication (power 250 W, frequency 40 kHz) in 10 mL of 70% aqueous methanol for 0.5 h. The mixtures were centrifuged at 12,000 rpm for 10 min at room temperature. Finally, the supernatants were combined prior to injection into a UV–vis spectrophotometer.

#### Preparation of the chromogenic solution for UV–vis spectroscopy

The test solution (0.1 mL) was accurately pippeted and added to a 10 mL volumetric flask. Then, 0.5 mL of 10% KOH was accurately pipetted and added to the same volumetric flask; this solution was left at 25 °C for 5 min. The solution mixture was then diluted with 70% aqueous ethanol and shaken well.

#### Preparation of the blank solution for UV–vis spectroscopy

KOH solution (0.5 mL; 10%) was accurately pipetted, added to a 10 mL volumetric flask, diluted with 70% aqueous ethanol, and shaken well.

### Instrumentation and measurement conditions

#### HPLC–PDA detection conditions

HPLC was performed using a Waters e2695 Alliance liquid chromatograph system (Waters, Milford, MA, USA) and a Waters 2998 photodiode array (PDA) detector (Waters, Milford, MA, USA). Licorice extracts were chromatographically separated using a Shiseido Capcell Pak MG C_18_ (4.6 mm × 250 mm, 5 µm; Tokyo, Japan) operating at 40 °C.

A liquid chromatography (LC) gradient was developed using a mobile phase consisting of solvent A (0.1% phosphoric acid/water) and solvent B (acetonitrile). The flow rate was set at 1.0 mL/min with the following gradient elution: 5–95% B (0–60 min), 95% B (60–65 min), 95–5% B (65–65.2 min); the elution was held at 5% B for 9.8 min to equilibrate the column. The sample injection volume was 10 µL, and the total analysis run time was 75 min. The PDA detection wavelengths were 250 and 330 nm.

#### UV–vis spectroscopy conditions

UV–vis spectroscopy was performed using a UV2700 UV–vis spectrophotometer (Shimadzu, Kyoto, Japan) operating at a 337 nm wavelength.

### Licorice appearance characterization

#### Length measurement

In each batch, the lengths of 30 licorice samples were randomly measured using a tape measure to obtain the average sample length.

#### Diameter measurement

In each batch, the diameters of 30 licorice samples were randomly measured using vernier callipers 2 cm below the reed head to obtain the average sample diameter.

#### Weight measurement

In each batch, 10 samples were randomly weighed using an ME 303 electronic balance (Mettler Toledo, Zurich, Switzerland) twice to obtain the average sample weight.

### Cell cultures and treatments

Murine macrophage (RAW264.7) cells, a widely used model for in vitro macrophage and inflammatory cascade studies, were obtained from the China National Collection of Authenticated Cell Cultures (Shanghai, China). The cells were maintained at 37 °C under 5% CO_2_ in Dulbecco’s modified Eagle’s medium (DMEM) supplemented with 10% foetal bovine serum (FBS). RAW264.7 cells were treated with 1 µg/mL lipopolysaccharide (LPS; Sigma–Aldrich; Merck KGaA, Darmstadt, Germany) for 24 h at 37 °C and were then rinsed with phosphate-buffered saline (PBS) and stimulated with LPS (1 µg/mL) to induce pyroptosis. The conditioned cells were collected to measure protein expression levels by enzyme-linked immunosorbent assay (ELISA) and western blot analysis.

### ***ELISA for detecting cytokine***

The culture medium was removed from the culture plate, the cells were digested with trypsin, and culture medium was added. The cells on the culture plate were washed with deionized water. The cell suspension was collected into a centrifuge tube and centrifuged at 1000 × g for 5 min at 2–8 °C. The culture medium was removed, and the cells were washed three times with prechilled PBS. The cells were resuspended with an appropriate amount of prechilled PBS. The sample was frozen and thawed 3 times under conditions of − 80 °C and room temperature to completely lyse the cells. The sample was centrifuged at 1500 × g for 10 min at 2–8 °C to remove cell debris, and the supernatant was collected [8–11].

Interleukin (IL)-1β and tumour necrosis factor (TNF)-α expression levels in the treated cell supernatants were measured via ELISA assays (IL-1β: Mouse: ml063132-C; TNF-α: Mouse: ml002095-C; Enzyme-linked Biotechnology Co., Ltd., Shanghai, China) according to the manufacturer’s instructions.

### Western blot analysis

RAW264.7 cell protein expression was detected using western blot analysis. Cells (1.6 × 10^5^/well) were plated overnight and then treated with the indicated bioactive compound concentrations. After 1 h, 1 μg/mL LPS was added. Then, the supernatant and precipitated cells were collected 24 h later. Immunoblotting was performed using antibodies against targeted proteins, including AKT1 (1:2,500; ab89402; Abcam, Cambridge, MA, USA), p-AKT1 (1:5,000; ab81283; Abcam, Cambridge, MA, USA), PI3K (1:1,000; 4249; Cell Signaling Technology, Inc., Danvers, MA, USA), and p-PI3K (1:1,000; bs-3332R; Bioss Antibodies, Biotechnology, Inc., Beijing, China). The blots were developed using an enhanced chemiluminescence kit (ECL, Amersham Biosciences, Buckinghamshire, UK) and were measured using a luminescent image analyser (LAS-3000, Fuji Photo Film Co., Ltd., Japan).

### Data processing

One-way analysis of variance (ANOVA), Student’s *t* test, and correlation analyses were performed using IBM SPSS Statistics 23.0 (IBM Corp., Armonk, NY, USA). Principal component analysis (PCA) and orthogonal partial least squares discriminant analysis (OPLS-DA) were performed using SIMCA^®^ software, version 13.0 (Umetrics, Umea, Sweden). Support vector machine (SVM) was performed using MATLAB^®^ software (MathWorks, Inc., Natick, MA, USA). HPLC fingerprint similarity was analysed using ChemPattern^®^ software, version 2017Pro (Chemmind, Beijing, China).

## Results and discussion

### Establishment of the traditional trait-based quality index

Licorice root has been used widely in TCM for ~ 4000 years, and the TCM quality has been traditionally evaluated mainly based on sensory characterization [12]. In this study, the licorice root length, diameter, and weight were the main traditional quality evaluation characteristics. To identify the quality-attribute-related appearance characterization indices, 80 licorice sample batches grown for different numbers of years in different production areas and harvested in different seasons were analysed using the method described in "Licorice appearance characterization"Section. The measurements are summarized in Additional file 1: Table S2.

#### One-way ANOVA and t test

The appearance characterization indices were analysed for licorice grown for different numbers of years in different production areas and harvested in different seasons. The licorice grown in traditional and main modern production areas exhibited significantly different diameters and weights, and the licorice grown for different numbers of years had significantly different lengths, diameters, and weights. The licorice harvested in different seasons had significantly different diameters and weights (*P* < 0.05), as listed in Additional file 1: Table S3. The statistical analysis results showed that the diameter and weight indices of licorice grown in different years and production areas and harvested in different seasons were significantly different and could therefore be used as quality indices to evaluate potential licorice appearance traits.

#### Correlation analysis

To further identify indices for evaluating licorice quality based on quality-attribute-related appearance traits, correlations between licorice diameter and weight were separately investigated for licorice grown in different production areas for different numbers of years and harvested in different seasons. The results are listed in Additional file 1: Table S4. According to the correlation analysis results, since licorice diameter and weight were significantly correlated for licorice grown for different numbers of years in different production areas and harvested in different seasons, either could be selected as an appearance characterization index for evaluating licorice quality. Because licorice diameter, one of the most important appearance traits traditionally used to evaluate licorice quality, was readily quantifiable and easily detected, it was used as the quality-attribute-related appearance characterization index for evaluating licorice quality.

### Establishment of the chemical composition-based internal quality index

#### Key-quality-attribute-based HPLC fingerprint index

##### HPLC fingerprint validation method

To ensure that the established HPLC method could be applied to analyse HPLC fingerprint similarity, the HPLC fingerprint precision, stability, and repeatability were validated using sample S81, which met the Chinese-Pharmacopoeia-based national drug standard.

###### Precision

The same sample was consecutively injected 6 times. In the HPLC fingerprint recorded at 250 nm, the relative retention time and peak area were calculated based on the licorice saponin G2 reference peak for each common fingerprint peak; the relative retention time relative standard deviation (RSD) was < 0.5% for each common peak and that the relative peak area RSD was < 3.0%. Additionally, in the HPLC fingerprint recorded at 330 nm, the relative retention time and peak area were calculated based on the isoliquiritin reference peak for each common fingerprint peak; the relative retention time RSD was < 0.5% for each common peak and that the relative peak area RSD was < 3.0%.

###### Stability

The same sample was injected at 0, 2, 4, 8, 12, 16, 20, and 24 h. In the HPLC fingerprint recorded at 250 nm, the relative retention time and peak area were calculated based on the licorice saponin G2 reference peak for each common fingerprint peak; the relative retention time RSD was < 0.5% for each common peak and that the relative peak area RSD was < 3.0%. Additionally, in the HPLC fingerprint recorded at 330 nm, the relative retention time and peak area were calculated based on the isoliquiritin reference peak for each common fingerprint peak; the relative retention time RSD was < 0.5% for each common peak and that the relative peak area RSD was < 3.0%.

###### Repeatability

Six replicate samples were prepared from the same licorice batch according to the method described in "Licorice appearance characterization" Section. In the HPLC fingerprint recorded at 250 nm, the relative retention time and peak area were calculated based on the licorice saponin G2 reference peak for each common fingerprint peak; the relative retention time RSD was < 0.3% for each common peak and that the relative peak area RSD was < 2.9%. Additionally, in the HPLC fingerprint recorded at 330 nm, the relative retention time and peak area were calculated based on the isoliquiritin reference peak for each common fingerprint peak; the relative retention time RSD was < 0.3% for each common peak and that the relative peak area RSD was < 3.0%.

##### HPLC fingerprint similarity analysis

**Fig. 1:**
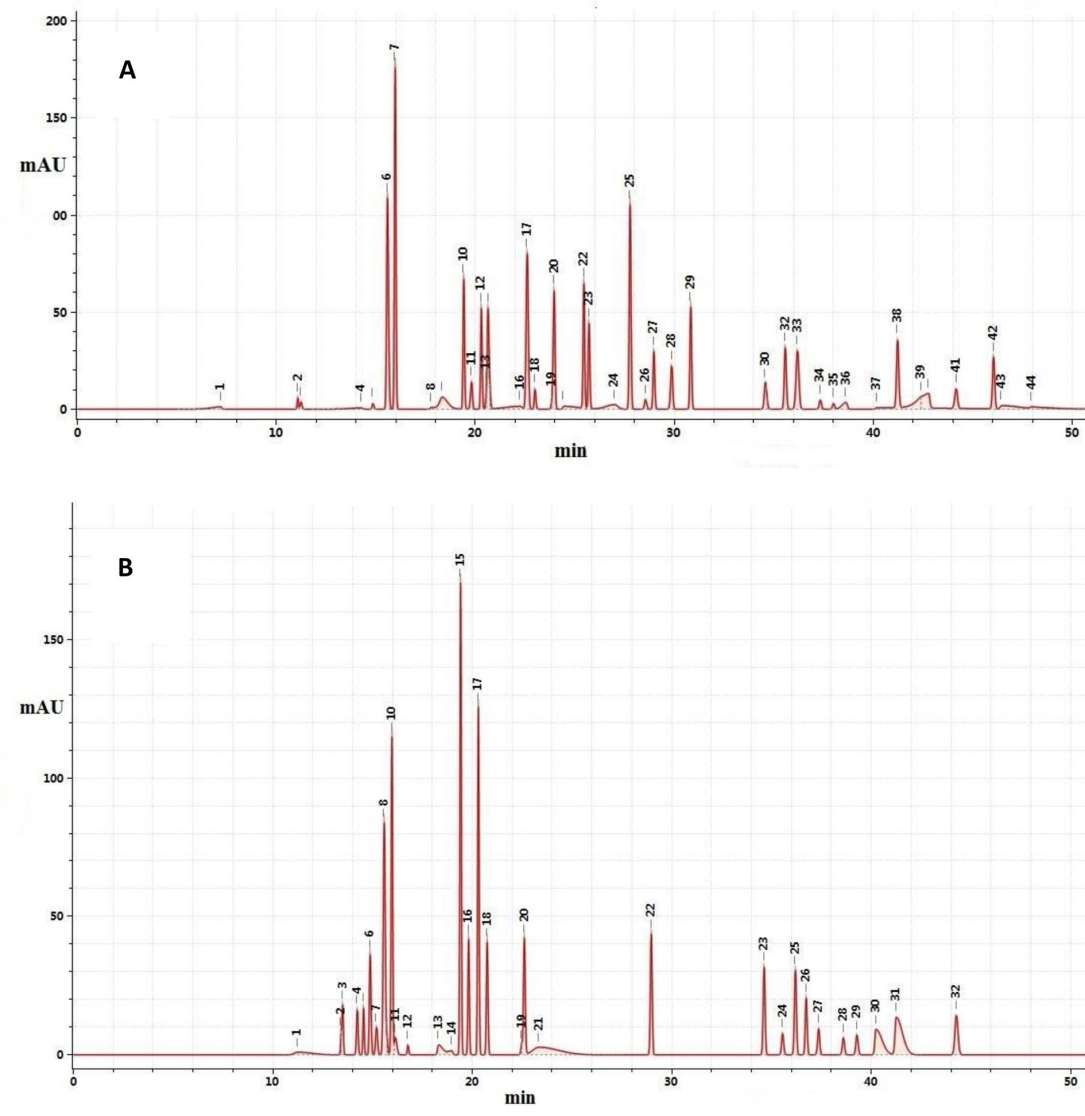
Common licorice HPLC fingerprint patterns recorded at **a** 250 and **b** 330 nm. At 250 nm, peak 6 is liquiritin apioside, peak 7 is liquiritin, peak 10 is isoliquiritin apioside, peak 12 is isoliquiritin, peak 13 is neoisoliquiritin, peak 17 is liquiritigenin, peak 25 is licoricesaponin G2, peak 27 is isoliquiritigenin, peak 28 is formononetin, peak 30 is glycycoumarin, peak 32 is semilicoisoflavone B, peak 34 is licoisoflavone A, and peak 38 is licoisoflavone B. At 330 nm, peak 6 is violanthin, peak 8 is liquiritin apioside, peak 10 is liquiritin, peak 15 is isoliquiritin apioside, peak 17 is isoliquiritin, peak 18 is neoisoliquiritin, peak 20 is liquiritigenin, peak 22 is isoliquiritigenin, peak 23 is glycycoumarin, peak 24 is semilicoisoflavone B, peak 27 is licoisoflavone A, peak 28 is glycyrol, peak 30 is licoflavonol, and peak 31 is licoisoflavone B

To investigate whether licorice grown for different numbers of years in different places of origin and harvested in different seasons exhibited different HPLC fingerprints, the test solution prepared according to the method described in "Preparation of the sample solutions for HPLC–PDA detection" Section and detected according to the method described in "HPLC–PDA detection conditions" Section was used to record chromatograms at 250 and 330 nm. The angle cosine method was used to calculate the similarity between the HPLC fingerprints recorded for licorice grown for different numbers of years in different production areas and harvested in different seasons and the common licorice HPLC fingerprint reference pattern, as shown in Fig. 1.

##### One-way ANOVA and *t* test analyses of licorice HPLC fingerprint similarity

The HPLC fingerprints recorded at 330 and 250 nm were significantly different for licorice samples grown for different numbers of years in different production areas and harvested in different seasons (*P* < 0.05); thus, the HPLC fingerprints recorded at 250 and 330 nm were identified as internal quality evaluation indices. The measurement results are summarized in Additional file 1: Table S5.

#### Total flavonoid index determined based on the key quality attributes

##### Analysis of the total licorice flavonoid contents

To investigate the different total flavonoid contents of licorice grown for different numbers of years in different production areas and harvested in different seasons, test solutions were prepared according to the methods described in "Preparation of the sample solutions for UV–vis spectroscopy" – "Preparation of the blank solution for UV–vis spectroscopy" Sections, and a reference solution was prepared according to the method described in "Solution preparation for standardizing (UV–vis) absorption spectra" Section. The total sample flavonoid concentrations were determined using the method described in "UV–vis spectroscopy conditions" Section. The measurement results are summarized in Additional file 1: Figure S1.

##### One-way ANOVA and *t* test analyses of the total licorice flavonoid contents.

SPSS^®^ 23.0 software was used to statistically analyse the total licorice flavonoid contents. The results showed that the total flavonoid contents of licorice samples grown for different numbers of years in different production areas and harvested in different seasons were significantly different (*P* < 0.05) and identified the total licorice flavonoid content as an internal quality evaluation index. The measurements are summarized in Additional file 1: Table S6.

#### Licorice extract contents determined based on the key quality attributes

To investigate the relationship between licorice extract contents and key quality attributes, licorice was extracted using water and 50% aqueous ethanol according to the relevant methods described in the Chinese Pharmacopoeia (2020 edition) [6]. The results were analysed using one-way ANOVA and *t* tests for both water- and alcohol-soluble licorice extracts grown for different numbers of years in different production areas and harvested in different seasons.

The statistical analyses showed that the alcohol-soluble licorice extract contents were significantly different (*P* < 0.05) for licorice grown in different production areas for different numbers of years and harvested in different seasons, while the water-soluble licorice extract contents were negligibly different for licorice grown in different production areas (*P* > 0.1). To investigate the correlation between the comprehensive licorice quality and the key quality attributes, the alcohol-soluble extract was identified as an internal quality evaluation index. The measurements are summarized in Additional file 1: Figure S2, and Table S7.

#### Chemical composition indices determined based on the key quality attributes

In our previous study [7], ultrahigh-performance liquid chromatography-quadrupole time-of-flight mass spectrometry (UHPLC-QTOF/MS/MS) and ultrahigh-performance liquid chromatography-triple quadrupole mass spectrometry (UHPLC-TQ-MS/MS) were used to analyse 158 batches of licorice grown for different numbers of years in different production areas and harvested in different seasons. We identified a panel consisting of 17 compounds that could be used as indices to evaluate the sample contents. Then, 158 sample batches were analysed using HPLC–PDA detection to determine the 17 compound contents. Validation of the HPLC determination method was completed in previous studies [7]. All analytes showed excellent linearity (R^2^ ≥ 0.9994) over the test concentration range. The LOD and LOQ values were in the range of 0.0061–0.0628 µg/mL and 0.0203–0.2092 µg/mL, respectively. The average recovery rate of the compounds was in the range of 91.19–101.25% (relative standard deviation, RSD ≤ 2.18%). The RSD values of the compound peak areas detected by the stability, precision, and repeatability tests were less than 2.92%, 1.00%, and 2.33%, respectively. Based on these results, the method was accurate and validated. Finally, multiple multivariate statistical analyses (one-way ANOVA, *t* test, PCA, and OPLS-DA) were performed to identify the sample TCM-quality-attribute-related chemical composition indices. Based on the VIP scores (VIP score ≥ 1.0) in OPLS-DA models, the results indicated that licorice saponin G2, glycyrrhizic acid, formononetin, violanthin, liquiritin apioside, liquiritin, isoliquiritin apioside, isoliquiritin, neoisoliquiritin, isoliquiritin isomer, liquiritigenin, semilicoisoflavone B, glycyrol, licoflavonol, and licoisoflavone B were suitable TCM-quality-attribute-related chemical composition indices.

### Establishment of a pharmacological activity-based quality index

In our previous study [13], the action mechanisms underlying licorice pharmacological effects were analysed using network pharmacology, and different bioactive compounds were simultaneously classified in licorice. The major licorice bioactive compounds were identified using UHPLC-QTOF-MS/MS. Liquiritin apioside, liquiritigenin, and isoliquiritin apioside were the main licorice bioactive components. Liquiritin and isoliquiritin apiosides consisted of the genes involved in NF-κB, TNF, and IL-1β, while liquiritigenin consisted of the genes involved in PI3K/AKT. Glycyrrhizic acid was the main representative licorice chemical component and comprised the genes involved in NF-κB and TNF. All these genes play important roles in anti-inflammatory immune activity. Thus, the anti-inflammatory properties of liquiritin apioside, liquiritigenin, glycyrrhizic acid, and isoliquiritin apioside were determined using ELISA and western blot analysis.

In our study, liquiritin apioside, liquiritigenin, glycyrrhizic acid, and isoliquiritin apioside were identified as pharmacological activity indices. The sum of these four pharmacological-activity indices was used as the pharmacological-activity-based licorice quality index.

### Comprehensive method for evaluating the licorice quality

In "Establishment of the traditional trait-based quality index" – "Establishment of a pharmacological activity-based quality index" Sections, standardized production and authenticity were selected as the key factors to ensure the production of high-quality licorice. We examined multiple quality indicators strongly correlated with the key licorice quality attributes (e.g., genuine production area, number of years of growth, and harvest season). In our study, the biological evaluation of licorice was carried out to identify pharmacological activity indices, and the quality-attribute-based evaluation was performed to identify quality indicators, including the traditional sensory-characterization-based licorice diameter index and other indices based on internal chemical components: alcohol-soluble extracts; total flavonoid contents; HPLC fingerprints recorded at 250 and 330 nm; contents of licorice saponin G2, glycyrrhizic acid, formononetin, violanthin, liquiritin apioside, liquiritin, isoliquiritin apioside, isoliquiritin, neoisoliquiritin, isoliquiritin isomer, liquiritigenin, semilicoisoflavone B, glycyrol, licoflavonol, and licoisoflavone B; and a pharmacological-activity-based quality index comprised the sum of the contents of glycyrrhizic acid, liquiritin apioside, liquiritigenin, and isoliquiritin apioside pharmacological activity indices.

These 21 quality indices are suitable candidates for establishing a method for grading licorice quality. Among the 282 batches of samples collected, 189 batches of sample quality met the Chinese-Pharmacopoeia-based national drug standards, which were used to establish a quality classification model. Considering the genuine production area, number of growth years, and harvest season as authentic quality evaluation factors, 189 licorice batches grown in different production areas for different numbers of years and harvested in different seasons were categorized as follows: 52, 92, and 45 batches of premium, first-class, and second-class samples, respectively. The measurement results are summarized in Additional file 1: Figure S3.

#### Development of the multivariate analysis model for grading licorice quality

**Fig. 2:**
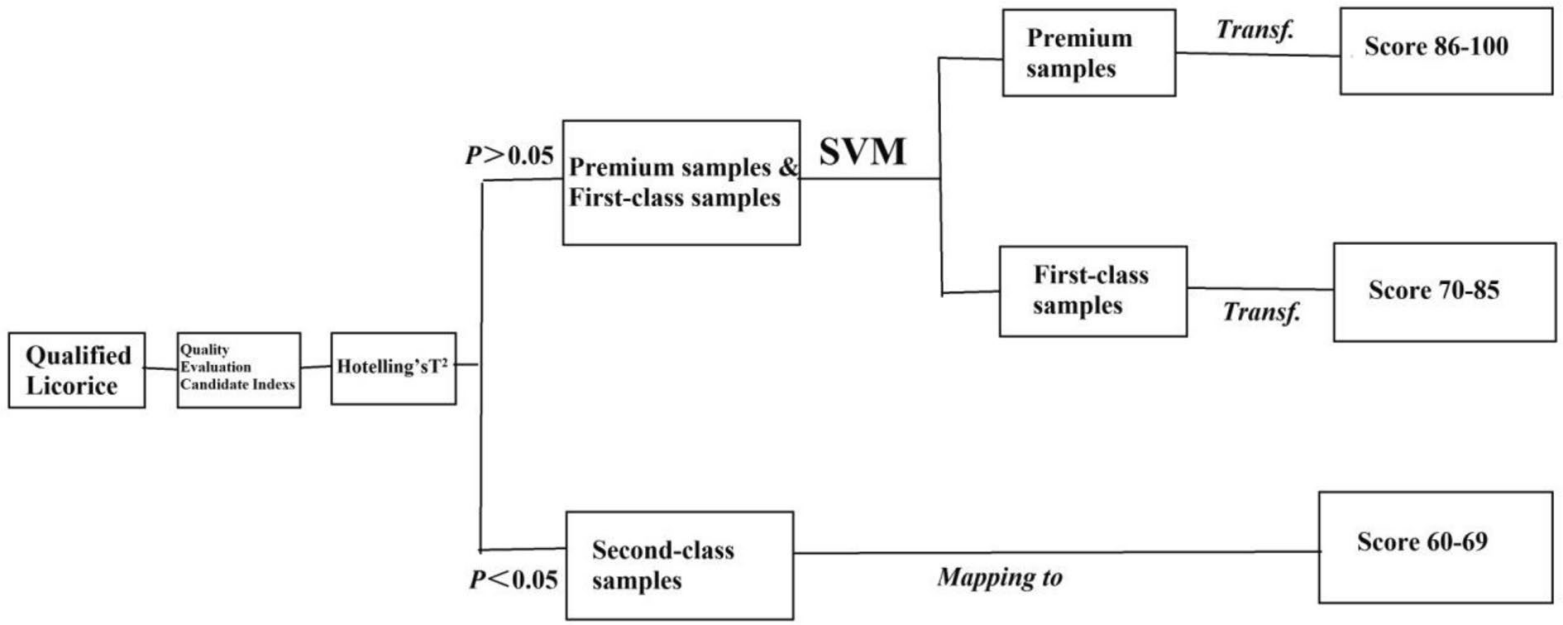
Model establishment process

Licorice is a complex multicomponent TCM system, and many key factors closely related to quality attributes, such as the genuine production area, number of growth years, and harvest season, further complicate comprehensive licorice quality evaluation. Owing to unique advantages such as nonlinear classification, small sample requirements, and high-dimensional pattern recognition, SVMs can facilitate comprehensive licorice quality evaluation [14–16]. Therefore, SVM was used to establish a method for grading licorice quality. Because SVMs operate based on binary classification models, licorice samples were initially classified according to three quality levels. Therefore, prior to SVM modelling, a sample confidence boundary was established as the basis for determining second-class samples, and the SVM was subsequently used to predict premium and first-class samples. Sample batches (189) were used as the research object, 21 candidate indices were used to establish the model for grading licorice quality, and a computer model was established using SVM analysis to predict the TCM quality composite evaluation index (TCM QCEI). The model establishment process is shown in Fig. 2. The research results were determined as follows:

***Step 1.*** By optimizing the grid, the radial basis function (RBF) SVM parameters were optimized in ranges from 10^−5^ to 10^5^ and from e^−5^ to e^5^. The optimal model parameters *C* and *γ* were 7.56 and 510.89, respectively, and the tenfold cross-validation accuracy was 91.56%.

***Step 2.*** The model parameters C and γ were unchanged, one variable was removed at a time, and the change value of the difference of the Jacobi matrix (DJ) was calculated.

***Step 3.*** The contributions of variables were sorted based on DJ value. Nine variables with relatively large DJ values were selected as the key quality parameters to retrain the model. Model parameters *c* and *γ* were 33.71 and 19634, respectively. The tenfold cross validation accuracy was 90.26%. Licorice diameter, total flavonoid content, similarities of HPLC chromatogram fingerprints recorded at 250 and 330 nm, contents of liquiritin apioside, liquiritin, glycyrrhizic acid, and liquiritigenin, and pharmacological activity quality index were identified as the key indices for constructing the model for evaluating licorice quality, and their contribution rates were proportionally weighted in the model. The contributions of the 21 candidate indicators to the SVM-based licorice-quality prediction model are shown in Table 1 and Additional file 1: Figure S4.

**Table 1:** Contributions of the 21 candidate indices to the SVM-based prediction model

Index	Contribution (%)	Index	Contribution (%)	Index	Contribution (%)	
Licorice saponin G2	0.94	Liquiritigenin	8.01	Licoflavonol		2.55 × 10^−2^
Glycyrrhizic acid	4.86	Violanthin	2.06 × 10^−2^	Alcohol-soluble extract		1.15
Formononetin	3.17 × 10^−2^	Isoliquiritin apioside	0.44	Diameter		12.73
Semilicoisoflavone B	3.33 × 10^−2^	Isoliquiritin isomer	3.39 × 10^−2^	Pharmacological activity index		14.52
Licoisoflavone B	0.72	Isoliquiritin	0.51	Total flavonoid content		26.14
Liquiritin apioside	4.83	Neoisoliquiritin	0.23	HPLC fingerprint chromatogram similarity (250 nm)		2.18
Liquiritin	20.07	Glycyrol	5.00 × 10^−3^	HPLC fingerprint chromatogram similarity (330 nm)		2.52

**Fig. 3:**
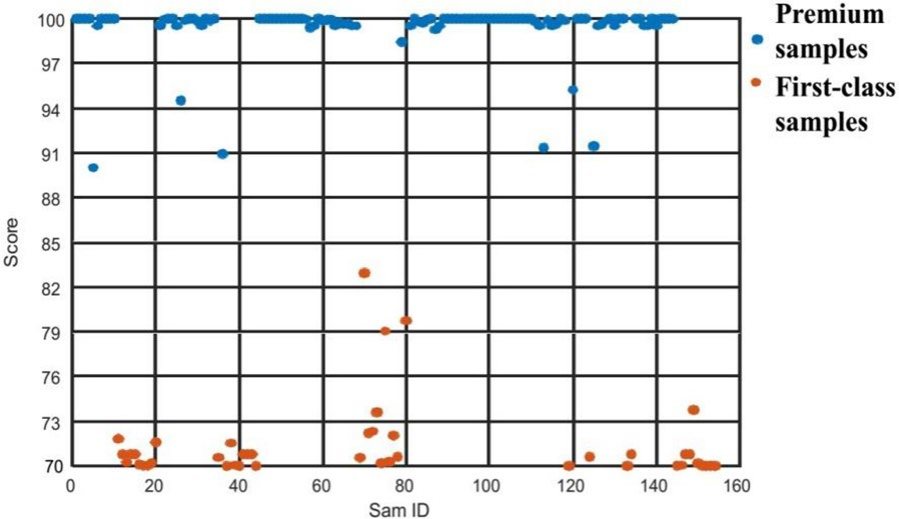
SVM calculated TCM QCEI classification diagram

***Step 4.*** Based on the training set sample scores, the model posterior probability was fitted using an S-type function to enlarge the model scale. The comprehensive score was estimated based on tenfold cross validation; the premium and first-class sample QCEI scores (in ranges 86–100 and 70–85, respectively) are shown in Fig. 3.

#### Validation of the licorice quality grading model preliminarily based on anti-inflammatory pharmacological effects

In this study, an RBF SVM and a TCM QCEI evaluation index were used to establish a model for grading licorice quality. The tenfold model cross-validation accuracy rate was 90.26%. To further confirm the model rationality, an in vitro cell model was used to preliminarily verify the pharmacological activities of different licorice quality grades.

According to the network pharmacology results obtained in our previous study, the main licorice bioactive component genes greatly contributed to anti-inflammatory immune activity [13]. In this study, to determine whether different licorice quality grades differentially reduced the inflammatory response, TNF-α and IL-1β levels were measured in RAW 264.7 cells via ELISA analysis. Premium and first-class licorice batches, as determined based on TCM QCEI scores, were selected as representative samples.

**Fig. 4:**
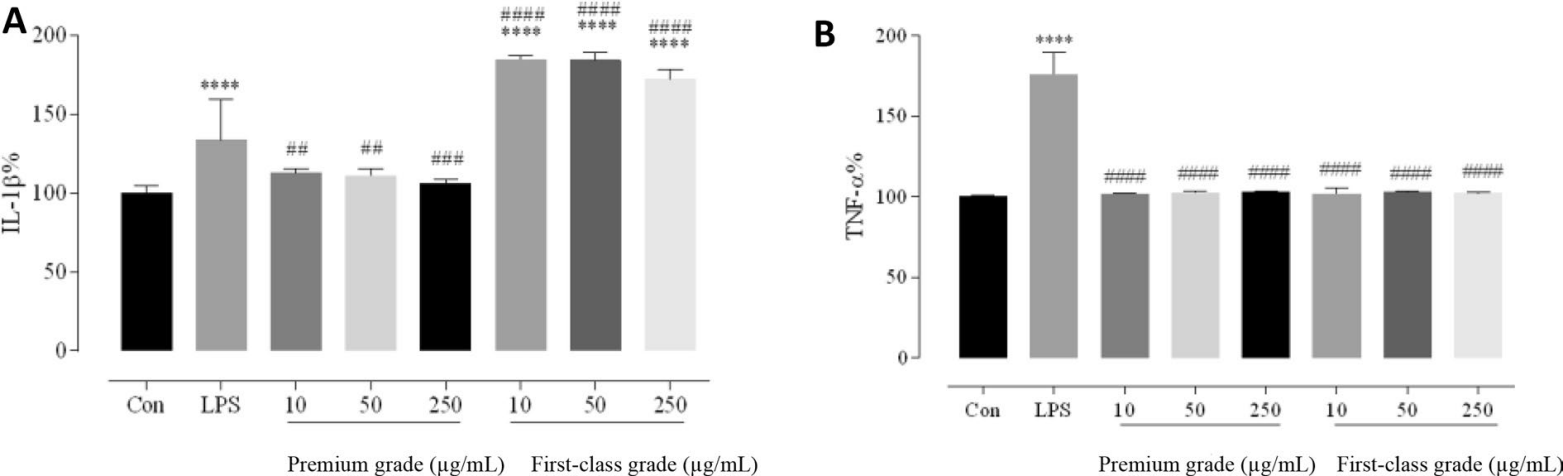
Effects of different licorice quality grades on reducing LPS-induced cytokine and chemokine production. Supernatants were collected for measuring **a** IL-1β and **b** TNF-α levels via ELISA. Data are presented as the mean ± standard mean error (*n* = 3). *P < 0.05, **P < 0.01, ***P < 0.001, and ****P < 0.001 *vs*. Con group; #*P* < 0.05, ##*P* < 0.01, ###*P* < 0.001, and ####*P* < 0.0001 *vs*. LPS group. IL, interleukin; LPS, lipopolysaccharide; TNF, tumour necrosis factor

In the in vitro experiment, the TNF-α and IL-1β levels in RAW 264.7 cells were significantly increased after LPS stimulation compared with their counterparts in the blank control (*P* < 0.0001). The pure LPS group showed a higher TNF-α level than those of the LPS groups treated with different licorice quality grades (*P* < 0.0001). The pure LPS group exhibited a higher IL-1β level than that of the LPS group treated with premium-grade licorice (*P* < 0.01) and a lower IL-1β level than that of the LPS group treated with first-class licorice (*P* < 0.0001); these results indicated that excessive LPS-induced TNF-α and IL-1β secretion could be reduced by different licorice quality grades and only premium-grade licorice, respectively (Fig. 4). Licorice chemical components are extremely complex. Some and other components exhibit anti-inflammatory and bidirectional regulatory effects, respectively. In this study, different licorice quality grades inhibited TNF-α but affected the IL-1β differently, indicating that the anti-inflammatory and bidirectional regulatory components could affect the TNF-α and IL-1β ratios differently. Therefore, different anti-inflammatory and bidirectional regulatory component contents could change TNF-α and IL-1β trends, and some chemical component contents in different licorice quality grades could be different and lead to different efficacies [17–19]. These results preliminarily indicated that all different licorice quality grades exhibited anti-inflammatory activity and that the inflammatory responses were likely better reduced by the premium-grade than by first-class licorice.

**Fig. 5:**
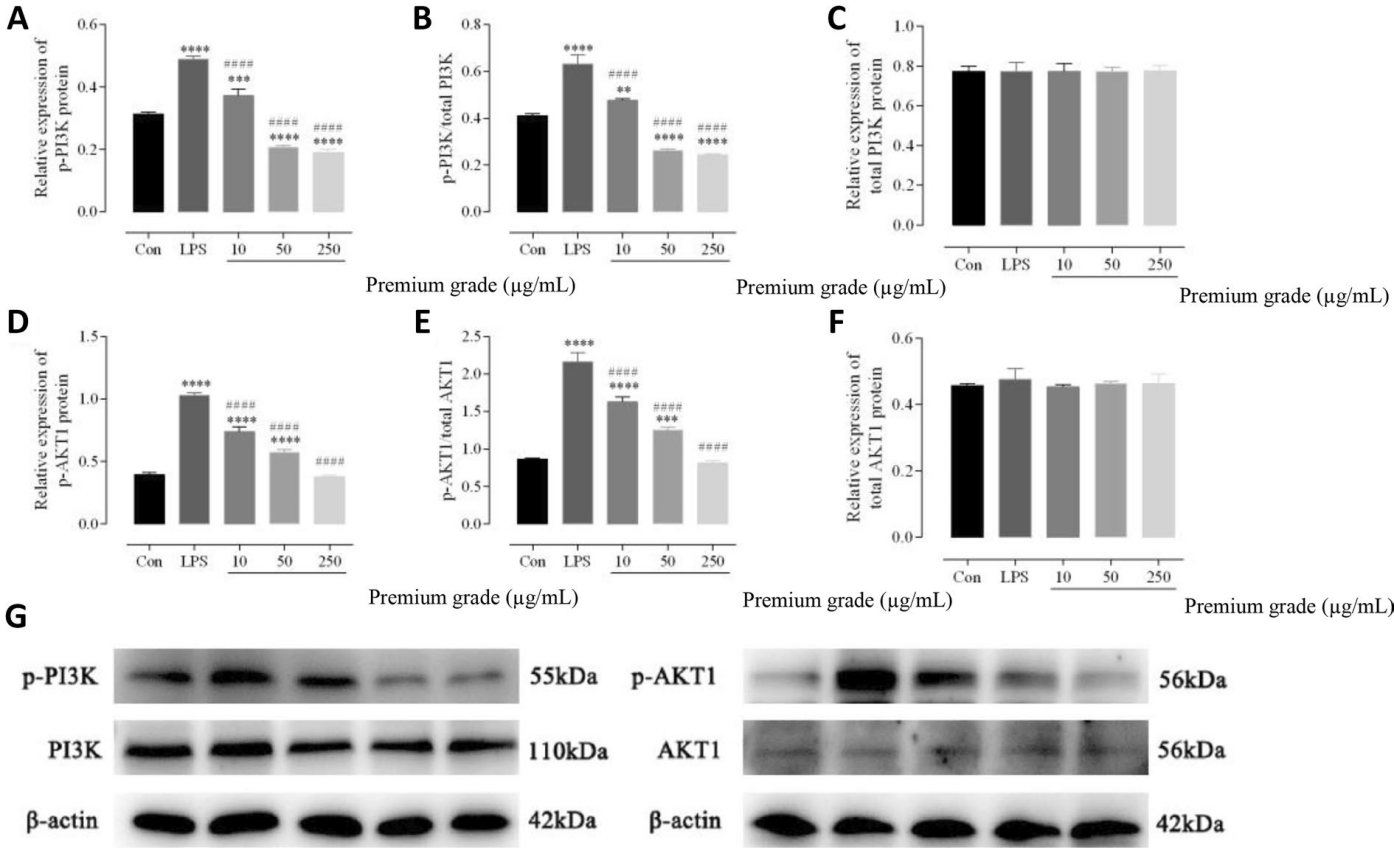
Effects of licorice on reducing the PI3K/AKT signalling pathway. PI3K and AKT protein levels were detected using **g** western blotting. Quantification of the relative **a** p-PI3K and **d** p-AKT1 protein expressions, **b** p-PI3K/PI3K and **e** p-AKT1/AKT1 ratios and total **c** PI3K and **f** AKT1 proteins. Data are presented as the mean ± standard mean error (*n* = 3). *P < 0.05, **P < 0.01, ***P < 0.001, and ****P < 0.001 *vs*. Con group; #*P* < 0.05, ##*P* < 0.01, ###*P* < 0.001, and ####*P* < 0.0001 *vs*. LPS group. LPS, lipopolysaccharide

To investigate the mechanisms underlying the anti-inflammatory activities of licorice, western blotting analysis was used to verify the regulatory effects of licorice. Western blotting showed that licorice markedly suppressed the PI3K/AKT signalling pathway mediated by p-PI3K (Fig. 5a), p-AKT1 (Fig. 5d), p-PI3K/PI3K (Fig. 5b), and p-AKT1/AKT1 (Fig. 5e) in the LPS-activated macrophages.

The PI3K/AKT signalling pathway greatly contributes to regulating signal transduction and biological processes, such as cell proliferation, apoptosis, metabolism, and angiogenesis [20, 21]. Our results preliminarily indicated that the main licorice bioactive components inhibited both TNF-α and IL-1β expression downstream through the PI3K/AKT signalling pathway and further demonstrated that licorice exhibited anti-inflammatory properties.

In our study, an in vitro cell model was used to preliminarily verify the pharmacological activities of different licorice quality grades. However, in future studies, more sample batches with different quality grades and more pharmacological models should be investigated to further confirm the rationality of TCM QCEI.

## Conclusions

In our study, traditional sensory characterization and modern standardized processes based on the production process and pharmacological efficacy evaluation were integrated for use in the assessment of TCM quality. Standardized licorice production and authenticity were used as the core elements to ensure that high-quality licorice was produced, and multiple quality indicators strongly correlated with the key licorice quality attributes (e.g., genuine production areas, number of growth years, and harvest season) were examined. Multidimensional quality evaluation indices were integrated with a machine learning model to identify key quality indices and their corresponding weight coefficients, to establish a multiweighted multi-index and comprehensive quality index and to construct a QCEI-based model for grading TCM quality.

For the first time, this study proposes a TCM QCEI based on appearance characterization, internal chemical composition quality, and pharmacological activity indices as a comprehensive multi-index to grade TCM quality and establishes a method for grading TCM quality. Additionally, this is the first study wherein different quality indicator weight contributions have been elucidated, a machine learning algorithm has been used to determine key quality evaluation indicators and their corresponding weight coefficients based on multidimensional research, a comprehensive model has been constructed to grade TCM quality, and TCM QCEIs have been calculated and applied to grade TCM quality. Our TCM QCEI integrates traditional appearance characterization and mainstream physical and chemical analyses and is correlated with pharmacological activities, thereby providing technical support for scientifically and objectively evaluating TCM quality.

## Supplementary Information

**Additional file 1.** **Figure S1:** Box plot of total flavonoids in licorice with different growth years, origins, and harvesting seasons. **Figure S2:** Box plot of water-soluble extract and alcohol-soluble extract in licorice with different growth years, origins,and harvesting seasons. **Figure S3:** Scatter plot of determination results of 21 candidate indicators in three grades of licorice. **Figure S4:** Line chart of the weight contribution of 21 candidate indicators in the quality evaluation model. **Table S1:** 282 batches of licorice sample information table. **Table S2:** Scatter plot of the measurement results of licorice appearance traits indicators. **Table S3:** Statistical analysis results of appearance traits indicators of licorice. **Table S4:** Correlation analysis of diameter and weight of licorice with different growth years, production areas, andharvesting seasons. **Table S5:** Statistical analysis of similarity in fingerprint of licorice with different growth years, place of origin, andharvesting seasons. **Table S6:** Statistical analysis of total flavonoids of licorice with different growth years, place of origin, and harvestingseasons.**Table S7:** Statistical analysis of water-soluble extracts and alcohol-soluble extracts of licorice with different growthyears, place of origin, and harvesting seasons.

T

## Data Availability

The datasets used and/or analysed during the current study are available from the corresponding author upon reasonable request.

## Abbreviations

ANOVA: Analysis of variance
DMEM: Dulbecco’s modified Eagle’s medium
ECL: Enhanced chemiluminescence
ELISA: Enzyme-linked immunosorbent assay
FBS: Foetal bovine serum
HPLC: High-performance liquid chromatography
IL: Interleukin
LPS: Lipopolysaccharide
LC: Liquid chromatography
MS: Mass spectrometry
NIFDC: National Institutes for Food and Drug Control
PBS: Phosphate-buffered saline
OPLS-DA: Orthogonal partial least squares discriminant analysis
PDA: Photodiode-array
PCA: Principal component analysis
QCEI: Quality composite evaluation index
QC: Quality control
Q-marker: Quality marker
RBF: Radial basis function
RSD: Relative standard deviation
SPSS®: Statistical package for the social sciences
SVM: Support vector machine
TCM: Traditional Chinese medicine
TNF: Tumour necrosis factor
UHPLC-QTOF-MS: Ultrahigh-performance liquid chromatography-quadrupole time-of-flight mass spectrometry
UHPLC-TQ-MS: Ultrahigh-performance liquid chromatography-triple quadrupole mass spectrometry
UV–vis: Ultraviolet–visible
DJ: The difference of the Jacobi matrix
VIP: Variable importance in the project
