# Supplementary figures and images for: A quality-comprehensive-evaluation-index-based model for evaluating traditional Chinese medicine quality
Source: Chin Med. 2023 Jul 28;18:89. doi: 10.1186/s13020-023-00782-0 (PMC10375775; doi:10.1186/s13020-023-00782-0)

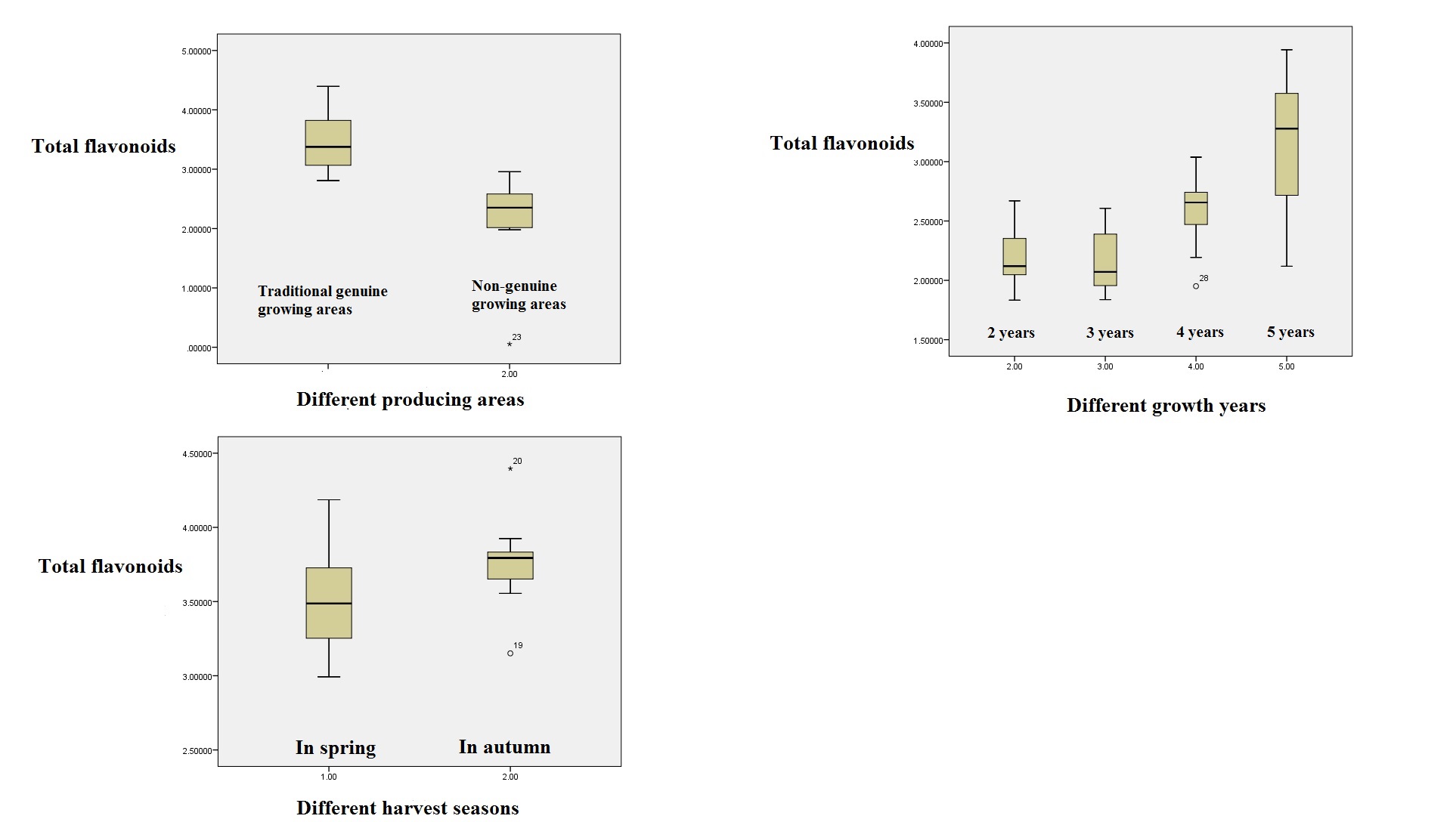

Supplement: Supplementary file 1 — Additional file 1. Figure S1: Box plot of total flavonoids in licorice with different growth years, origins, and harvesting seasons. Figure S2: Box plot of water-soluble extract and alcohol-soluble extract in licorice with different growth years, origins,and harvesting seasons. Figure S3: Scatter plot of determination results of 21 candidate indicators in three grades of licorice. Figure S4: Line chart of the weight contribution of 21 candidate indicators in the quality evaluation model. Table S1: 282 batches of licorice sample information table. Table S2: Scatter plot of the measurement results of licorice appearance traits indicators. Table S3: Statistical analysis results of appearance traits indicators of licorice. Table S4: Correlation analysis of diameter and weight of licorice with different growth years, production areas, andharvesting seasons. Table S5: Statistical analysis of similarity in fingerprint of licorice with different growth years, place of origin, andharvesting seasons. Table S6: Statistical analysis of total flavonoids of licorice with different growth years, place of origin, and harvestingseasons.Table S7: Statistical analysis of water-soluble extracts and alcohol-soluble extracts of licorice with different growthyears, place of origin, and harvesting seasons. [file 13020_2023_782_MOESM1_ESM.zip › Supplementary Fig.1-Be modified.jpg]

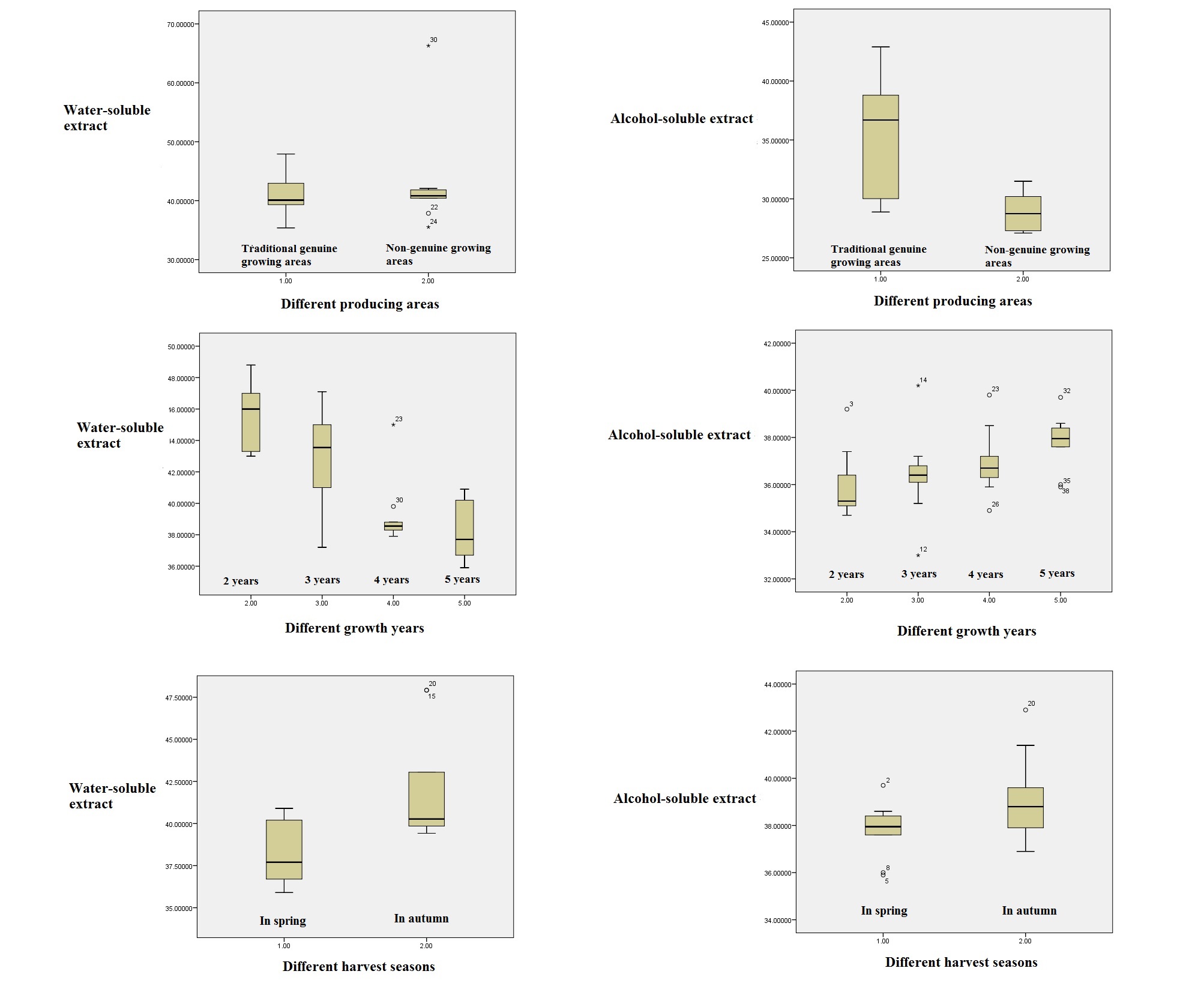

Supplement: Supplementary file 1 — Additional file 1. Figure S1: Box plot of total flavonoids in licorice with different growth years, origins, and harvesting seasons. Figure S2: Box plot of water-soluble extract and alcohol-soluble extract in licorice with different growth years, origins,and harvesting seasons. Figure S3: Scatter plot of determination results of 21 candidate indicators in three grades of licorice. Figure S4: Line chart of the weight contribution of 21 candidate indicators in the quality evaluation model. Table S1: 282 batches of licorice sample information table. Table S2: Scatter plot of the measurement results of licorice appearance traits indicators. Table S3: Statistical analysis results of appearance traits indicators of licorice. Table S4: Correlation analysis of diameter and weight of licorice with different growth years, production areas, andharvesting seasons. Table S5: Statistical analysis of similarity in fingerprint of licorice with different growth years, place of origin, andharvesting seasons. Table S6: Statistical analysis of total flavonoids of licorice with different growth years, place of origin, and harvestingseasons.Table S7: Statistical analysis of water-soluble extracts and alcohol-soluble extracts of licorice with different growthyears, place of origin, and harvesting seasons. [file 13020_2023_782_MOESM1_ESM.zip › Supplementary Fig.2-Be modified.jpg]

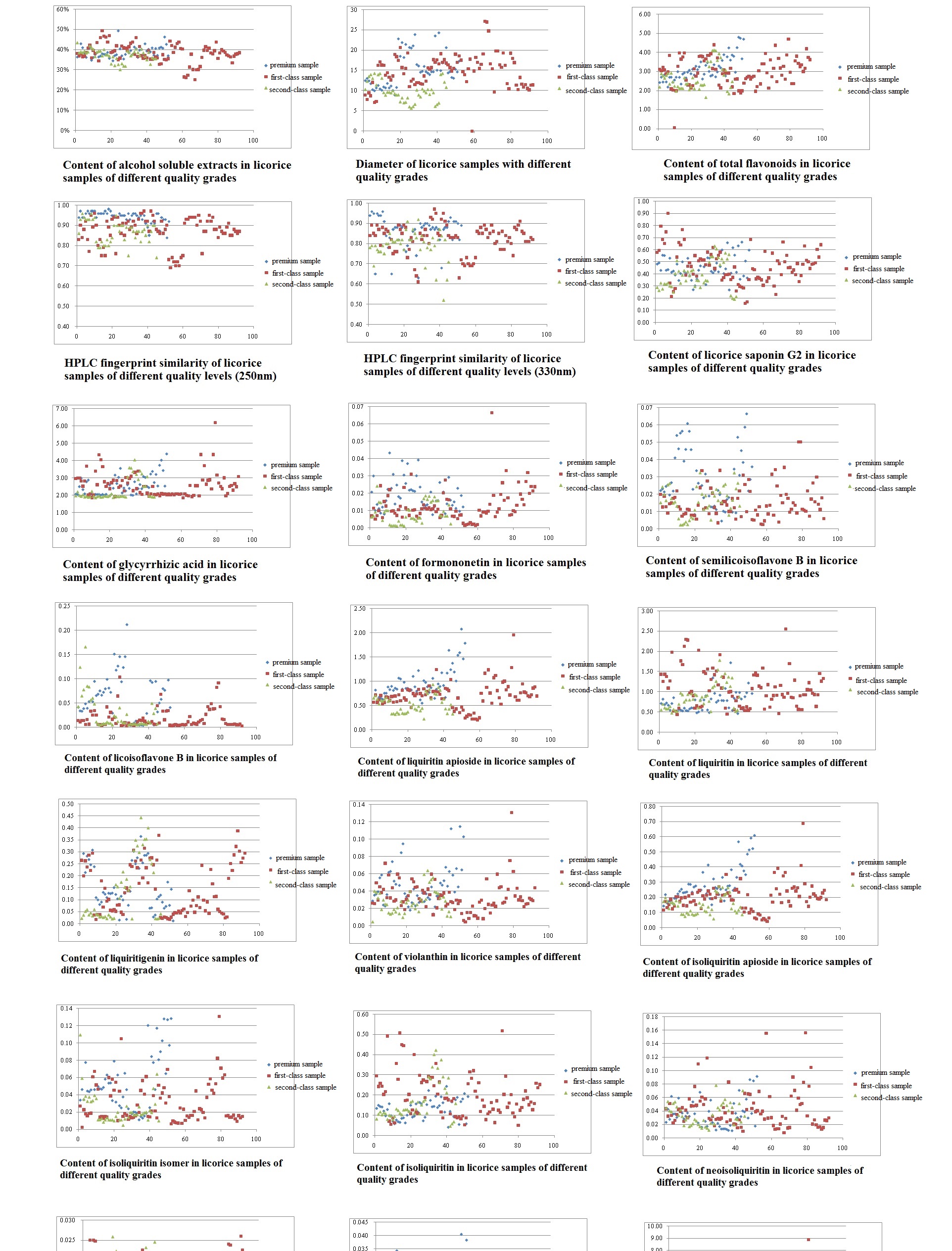

Supplement: Supplementary file 1 — Additional file 1. Figure S1: Box plot of total flavonoids in licorice with different growth years, origins, and harvesting seasons. Figure S2: Box plot of water-soluble extract and alcohol-soluble extract in licorice with different growth years, origins,and harvesting seasons. Figure S3: Scatter plot of determination results of 21 candidate indicators in three grades of licorice. Figure S4: Line chart of the weight contribution of 21 candidate indicators in the quality evaluation model. Table S1: 282 batches of licorice sample information table. Table S2: Scatter plot of the measurement results of licorice appearance traits indicators. Table S3: Statistical analysis results of appearance traits indicators of licorice. Table S4: Correlation analysis of diameter and weight of licorice with different growth years, production areas, andharvesting seasons. Table S5: Statistical analysis of similarity in fingerprint of licorice with different growth years, place of origin, andharvesting seasons. Table S6: Statistical analysis of total flavonoids of licorice with different growth years, place of origin, and harvestingseasons.Table S7: Statistical analysis of water-soluble extracts and alcohol-soluble extracts of licorice with different growthyears, place of origin, and harvesting seasons. [file 13020_2023_782_MOESM1_ESM.zip › Supplementary Fig.3-Be modified.jpg]

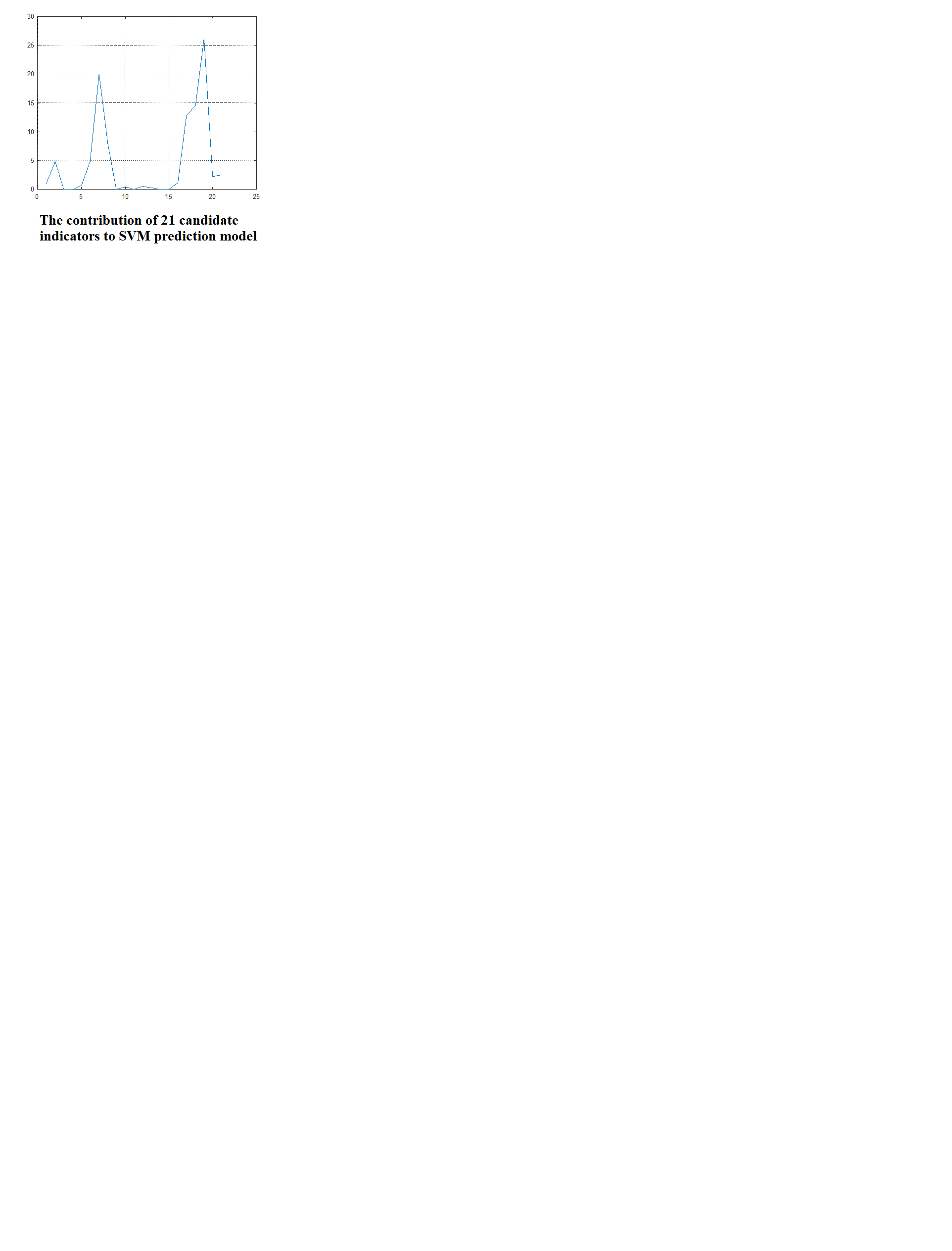

Supplement: Supplementary file 1 — Additional file 1. Figure S1: Box plot of total flavonoids in licorice with different growth years, origins, and harvesting seasons. Figure S2: Box plot of water-soluble extract and alcohol-soluble extract in licorice with different growth years, origins,and harvesting seasons. Figure S3: Scatter plot of determination results of 21 candidate indicators in three grades of licorice. Figure S4: Line chart of the weight contribution of 21 candidate indicators in the quality evaluation model. Table S1: 282 batches of licorice sample information table. Table S2: Scatter plot of the measurement results of licorice appearance traits indicators. Table S3: Statistical analysis results of appearance traits indicators of licorice. Table S4: Correlation analysis of diameter and weight of licorice with different growth years, production areas, andharvesting seasons. Table S5: Statistical analysis of similarity in fingerprint of licorice with different growth years, place of origin, andharvesting seasons. Table S6: Statistical analysis of total flavonoids of licorice with different growth years, place of origin, and harvestingseasons.Table S7: Statistical analysis of water-soluble extracts and alcohol-soluble extracts of licorice with different growthyears, place of origin, and harvesting seasons. [file 13020_2023_782_MOESM1_ESM.zip › Supplementary Fig.4.bmp]
